# Supplementary material for: Incidence, associated risk factors, and the ideal mode of delivery following preterm labour between 24 to 28 weeks of gestation in a low resource setting
Source: PLoS One. 2021 Jul 22;16(7):e0254801. doi: 10.1371/journal.pone.0254801 (PMC8297859; doi:10.1371/journal.pone.0254801)

**INCIDENCE, ASSOCIATED RISK FACTORS, AND THE IDEAL MODE OF DELIVERY FOLLOWING PRETERM LABOUR BETWEEN 24 TO 28 WEEKS OF GESTATION IN A LOW RESOURCE SETTING:**

BY: **DR HERBERT KAYIGA *MBChB, MMED OBS/GYN, MPH, GLOBAL HEALTH FELLOW***

**SUPERVISORS:**

1. **JOSAPHAT BYAMUGISHA, (*MMED OBS/GYN, PhD*)**

ASSOCIATE PROFESSOR,

MAKERERE UNIVERSITY, DEPARTMENT OF OBSTETRICS & GYNAECOLOGY,

1. **DR. ANNETTE NAKIMULI, (*MMED OBS/GYN, PhD*)**

ASSOCIATE PROFESSOR& CHAIR,

MAKERERE UNIVERSITY, DEPARTMENT OF OBSTETRICS &GYNAECOLOGY

**DECLARATION**

I declare that this is an original study and has never been submitted anywhere for any academic awards or publication.

**Dr. HERBERT KAYIGA**, MBChB, MPH, MMED Obs/Gyn (MAK)

Signature…………………………….. Date………………………………….

**SUPERVISORS**

1. **ASSOC. PROF ANNETTEE NAKIMULI,** MBChB, M.MED Obs/ Gyn (MAK), PhD

Associate Professor,

Makerere University- Department of Obstetrics and Gynaecology

Signature…………………………….. Date………………………………….

1. **ASSOC. PROF JOSAPHAT .K. BYAMUGISHA**, MBChB, M.MED Obs/ Gyn (MAK), PhD

Associate Professor,

.

Signature…………………………..Date……………………………………….

TABLE OF CONTENTS

[**DECLARATION** 36](#_Toc15986827)

[LIST OF ACRONYMS 39](#_Toc15986828)

[DEFINITION OF TERMS 40](#_Toc15986829)

[ABSTRACT 41](#_Toc15986830)

[INTRODUCTION: 42](#_Toc15986831)

[1.1 Background 42](#_Toc15986832)

[1.2 Problem Statement 43](#_Toc15986833)

[1.3 Justification 44](#_Toc15986834)

[1.4 Research Questions 45](#_Toc15986835)

[1.4.1 General Objective 46](#_Toc15986836)

[1.4.2 Specific Objectives 46](#_Toc15986837)

[2.0 LITERATURE REVIEW 46](#_Toc15986838)

[2.1 Preterm labour 46](#_Toc15986839)

[2.1.1 Definition 46](#_Toc15986840)

[2.1.2 Epidemiology 47](#_Toc15986841)

[2.1.3 Risk factors 47](#_Toc15986842)

[2.1.4 Etiology 50](#_Toc15986843)

[2.2 Diagnosis 50](#_Toc15986844)

[2.3 Management 53](#_Toc15986845)

[2.4.1 Management of Preterm labour between 24 to 28 weeks 55](#_Toc15986846)

[2.4.2 Below 24 weeks 56](#_Toc15986847)

[3.0 METHODS: 56](#_Toc15986848)

[3.1 Study Design 56](#_Toc15986849)

[3.2 Study Setting 56](#_Toc15986850)

[3.3 Study Period 57](#_Toc15986851)

[3.4 Case definition of study participants 57](#_Toc15986852)

[3.5 Inclusion criteria 57](#_Toc15986853)

[3.6 Exclusion Criteria 58](#_Toc15986854)

[3.8 Sample Size Calculation 58](#_Toc15986855)

[3.9 Outcome variables 59](#_Toc15986856)

[3.9.1 Primary Outcome 59](#_Toc15986857)

[3.10 DATA MANAGEMENT 59](#_Toc15986858)

[310.1 Data collection procedure 60](#_Toc15986859)

[3.10.2 Statistical analysis 60](#_Toc15986860)

[3.10.3 Quality Control 61](#_Toc15986861)

[3.11 Ethical Consideration 61](#_Toc15986862)

[3.12 Study Limitations 61](#_Toc15986863)

[3.13 Project Timeline and Plan 62](#_Toc15986864)

[4.0 REFERENCES 62](#_Toc15986865)

[5.0 WORK PLAN: 67](#_Toc15986866)

[6.0 BUDGET: 67](#_Toc15986867)

[APPENDIX I: MENTORSHIP PLAN 68](#_Toc15986868)

[APPENDIX II: CONSENT 68](#_Toc15986869)

[APPENDIX III: CONSENT FORM 71](#_Toc15986870)

[APPENDIX IV: LUGANDA CONSENT 72](#_Toc15986871)

[APPENDIX V: DATA COLLECTION TOOL 75](#_Toc15986872)

# LIST OF ACRONYMS

**C/S** Caesarean Section

**HDU** High Dependency Unit

**HIV** Human Immune Virus

**IUFD** Intrauterine Death

**MakCHS** Makerere University College of Health Sciences

**PROM** Premature Rupture of Membranes

**PPROM** Preterm Premature Rupture of Membranes

**SCU** Neonatal Special Care Unit

**SVD** Spontaneous vaginal delivery

# DEFINITION OF TERMS

**Conservative Management:**  Interventions undertaken to prolong a pregnancy.

**Latency**: Period from spontaneous membrane rupture to delivery.

**Preterm labour**: Spontaneous onset of contractions leading to progressive cervical effacement and dilatation occurring between 24 to 28 weeks of gestation.

**PROM:**  Spontaneous membranes rupture prior to onset of labour.

**PPROM**: Spontaneous membranes rupture after 24 but less than 37 weeks of gestation

**Prolonged PROM:** Spontaneous membranes rupture for over 24 hours prior to delivery.

**Viability**: Gestation age of more or equal to 28 weeks.

#

# ABSTRACT

**Background**: Preterm labour remains prevalent in low resource settings between 24 to 28 weeks of gestation. With advances in medicine, there’s evidence of survival after 24 weeks. The ideal mode of delivery remains unclear as there are no clear management protocols to streamline patient management in preterm labour occurring between 24 and 28 weeks of gestation.

We seek to determine the prevalence of preterm labour occurring between 24 to 28 weeks, identify its associated risk factors and determine the ideal mode of delivery in a low resource setting with the aim of streamlining patient care.

**Methods**: A descriptive hospital based cross-sectional study will be carried out to determine the prevalence, associated risk factors and ideal mode of delivery for preterm labour occurring between 24 to 28 weeks of gestation. We plan to follow up 383 women and their babies following preterm labour between 24 weeks to 28 weeks of gestation from admission to discharge from the hospital. The primary outcome will be preterm birth. Secondary outcomes will include neonatal and maternal infection, admission to the Neonatal Special Care Unit (SCU), perinatal mortality, length of stay in SCU, need for neonatal resuscitation, maternal wound disruption (vaginal or abdominal) and maternal death.

**Significance of Study**: The study will provide guidance for management of preterm labour occurring between 24 to 28 weeks in low resource settings. Findings from the study could improve neonatal outcomes, reduce the cesarean delivery rate, decrease maternal and neonatal complications, and shorten the decision to delivery interval for other patients who require emergency operative delivery by reducing the number of cesarean deliveries in these settings. The potential system improvements of decreasing the number of women awaiting cesarean and reducing hospital stay of mothers and infants could be profound. Patients at risk of preterm labour would be given timely referral to tertiary institutions to optimize pregnancy outcomes. Interventions tailored to prevent preterm labour would be developed thereby improving pregnancy outcomes in the subsequent pregnancies.

INTRODUCTION:

1.1 Background**:**

Preterm labour, which is the spontaneous onset of frequent contractions of significant intensity leading to progressive cervical effacement and dilatation before 37 weeks, complicates 5-10% of all pregnancies (1). Preterm labour leads to nearly half of all preterm deliveries (2). The incidence of preterm labour in Europe is between 5-11% (3). In the United States, the incidence of preterm labour is about 12% (4). The incidence of preterm labour in Sub-Saharan Africa is between 15-38% (4, 5). The chances of an African preterm baby dying from complications of prematurity are 12 times more than those of a European baby (5).

Globally 15 million preterm babies are born (6), 60% of these preterm babies are born in Sub-Saharan Africa and Asia. In Uganda, of the 1,665,000 annual births, 226,000 are born preterm of which 12,500 die from complications of prematurity(7). Prematurity contributes 38% of all the neonatal mortality, also the leading cause of under-5 year mortality in Uganda(8).

A review of the Kawempe General Hospital records in 2018 shows of the 24,526 deliveries, 2784 were prematures. Preterm labour occurring between 24 to 28 weeks also referred to as inevitable abortion, has contributed 74 out 326 abortions in the Emergency gynaecology unit from January to March 2019. Eighty-five percent of neonatal morbidity and 30% of the mortality in the Special Care Unit (SCU) results from prematurity, a sequel of preterm labour (9).

Whereas 85% of the preterm deliveries occur between 32 to 37 weeks(10), there’s a significant proportion of preterm deliveries occurring below 28 weeks. These are referred to as extreme preterm babies (11). In Uganda, these are considered as inevitable abortion, management is usually expectant and these preterm babies often die due inadequate pre-delivery preparation. Understanding the prevalence and risk factors leading to preterm labour from 24 to 28 weeks and ideal mode of delivery, will guide timely referral; optimize pregnancy outcomes and prevent reoccurrence in the subsequent pregnancies.

Though there is evidence that survival is minimal below 23 weeks, with advances in technology, corticosteroids, tocolytics and antibiotics, preterm babies can survive after 24 weeks(10). The ideal mode of delivery in gestation age between 24 to 28 weeks remains unclear in low resource settings. A significant number of mothers scheduled for caesarean section, deliver vaginally before accessing theatre.

It’s against this backdrop that we seek to explore the prevalence, associated risk factors and ideal mode of delivery for preterm delivery occurring between 24 to 28 weeks with the aim of streamlining patient care for those at risk to improve their pregnancy outcome.

1.2 Problem Statement:

Although preterm labour between 24 to 28 weeks at Kawempe General Hospital occurs often (2784/24,526), it’s associated factors and ideal mode of delivery remain controversial. When this labour occurs before 28 weeks of gestation following infections or any other pathology, the delivery plan is often very challenging. There are no standard protocols to guide patient management. Three patients of the 78, who have presented from January 2019 to March 2019 at Kawempe General Hospital with inevitable abortion, had sepsis presenting as a complication while on conservative management.

Mothers are operated for indications such as severe oligohydramnios, inability to monitor mothers with preterm labour, abnormal presentation or prior caesarean section scars. There is also fear of intra-cerebral hemorrhage especially when labour occurs before 32 weeks. Though some of these mothers are scheduled for cesarean delivery, there is usually a long queue awaiting the same intervention. A review of the Kawempe General Hospital records shows that of 24,526 deliveries that occurred in 2018, 6547 (27%) were by caesarean delivery. This is already above the recommended 10-15% caesarean rate by the WHO (12). The other patients waiting for caesarean delivery at times have more life threatening conditions yet can’t access this intervention due to the congested theatre queues.

Even before accessing theatre, some of these mothers actually deliver. Due to the long decision – delivery interval, their outcomes are sometimes poorer than if the decision to augment the labour had been instituted initially.

Since there is no clear management protocol guiding the delivery of preterm babies between 24 and 28 weeks of gestation at the hospital, this study sets out to explore whether the perinatal death rate among mothers with preterm labour between 24 and 28 weeks following vaginal or caesarean delivery, is the same or even better for vaginal route. If vaginal delivery is found to be as safe as caesarean delivery, then there will be evidence to justify its uptake in low resource settings. This will in turn reduce on the waiting time for cesarean delivery for those with more life threatening emergencies. We shall also seek to determine the prevalence and associated risk factors of preterm labour between 24 to 28 weeks with the overall aim of streamlining patient care in a low resource setting.

1.3 Justification:

Despite the fact that evidence now shows that with tocolytics, corticosteroid use and advances in neonatal care, survival after 24 weeks is possible, in Uganda deliveries before 28 weeks are still referred to as inevitable abortion. Care is mainly expectant.

According to Stewart et al (13), 47% of the babies survived between 26 to 28 weeks. Talley et al (14) reported that 100% of all preterm babies after 26 weeks survived in their study. There is a need to reconsider the current practice and seek evidence based options to optimize obstetric outcomes following preterm labour between 24 and 28 weeks of gestation in our local setting.

If there is evidence that vaginal delivery is as safe as or even safer than caesarean delivery for indications such as severe oligohydramnios, inability to monitor fetus in preterm labour, then its uptake will increase thereby reducing the waiting time for mothers with preterm labour in Kawempe General Hospital. The overall maternal hospitalization for mothers with preterm labour would reduce thereby reducing the overall cost of care. Women with more grave emergencies would access theatre in time with decongestion of the theatre lists. With reduced decision-to- delivery interval, perinatal morbidity and mortality would reduce, as there would be reduction in chorioamnionitis with reduced latency period.

It is to this end that this study ought to be carried out to compare the outcomes of vaginal versus caesarean mode of delivery following preterm labour with the aim of improving the overall patient care in a low resource setting such as Kawempe General Hospital. Determination of the prevalence and risk factors of preterm labour between 24 to 28 weeks will also avail information on how to mitigate its re-occurrence in the subsequent pregnancies.

1.4 Research Questions**:**

1. What is the prevalence and associated risk factors of preterm labour occurring between 24 and 28 weeks of gestation at Kawempe General Hospital, Kampala, Uganda?
2. What is the ideal mode of delivery for women with preterm labour between 24 and 28 weeks of gestation at Kawempe General Hospital Kampala, Uganda?

1.4.1 General Objective:

To determine the prevalence, associated risk factors and ideal mode of delivery for preterm labour occurring between 24 and 28 weeks of gestation at Kawempe General Hospital, Kampala, Uganda

1.4.2 Specific Objectives:

1. To determine the prevalence of preterm labour occurring between 24 and 28 weeks of gestation at Kawempe General Hospital, Kampala, Uganda
2. To determine risk factors associated with preterm labour occurring between 24 and 28 weeks of gestation at Kawempe General Hospital, Kampala, Uganda.
3. To determine the difference in pregnancy outcomes for women with preterm labour between 24 and 28 weeks of gestation who have vaginal versus caesarean delivery at Kawempe General Hospital Kampala, Uganda.

2.0 LITERATURE REVIEW**:**

2.1 Preterm labour**:**

2.1.1 Definition**:** Preterm labour, which is the spontaneous onset of frequent contractions of significant intensity leading to progressive cervical effacement and dilatation before 37 weeks, complicates 5-10% of all pregnancies (1). Preterm labour leads to nearly half of all preterm deliveries (2).

2.1.2 Epidemiology:

Globally 15 million preterm babies are born (6), 60% of these preterm babies are born in Sub-Saharan Africa and Asia. The incidence of preterm labour in Europe is between 5-11% (3). The incidence of preterm labour in Sub-Saharan Africa is between 15-38% (4, 5). The chances of an African preterm baby dying from complications of prematurity are 12 times more than those of a European baby (5).

In Uganda, of the 1,665,000 annual births, 226,000 are born preterm of which 12,500 die from complications of prematurity (5). Prematurity contributes 38% of all the neonatal mortality, and is also the leading cause of under-5 year mortality in Uganda(8).

- - 1. Risk factors:

There is evidence that the trends of the preterm labour have increased globally over the last 20 years yet little has been put in place to mitigate the rising incidence (5). Over 80% of the preterm labour occurs spontaneously. The causes of spontaneous preterm labour in over 50% of the cases remain unknown yet identification of these risk factors would help to mitigate this rising burden and optimize pregnancy outcomes (4, 15, 16). The following risk factors have been identified in other studies

**Socio-demographic characteristics**:

**Race**: The Black patients are more at risk of preterm labour than the white population (17).

**Age:** Extremes of age have been identified as a risk factor for preterm labour. According to the study done in Kenya, age less than 20 years was identified as a protective factor against preterm labour (p= 0.034) (5) but others studies have reported contradicting findings. According to Kozuki et al, women of less than 20 years had more chances of having preterm labour. This was mainly because of their prematurity, nativity of pregnancy and childbirth. Such women were also at risk of stress in pregnancy which could lead to high cortisol levels and catecholamine production. These hormones in turn could trigger production of placental corticotrophin releasing hormone which would ultimately lead to preterm labour (18). A similar finding was reported in Indonesia, that women less than 20 years were 1.586 times more at risk of preterm labour as compared to 20-35 year group (p= 0.004) (11).

**Parity**: According to Wagura et al (5), women with a parity of 4 and above were five times more likely to have preterm labour as compared to those with a parity less than 4 (p=0.019; OR 4.709). Other studies didn’t find any associated between parity and preterm labour (11).

**Socio-economic status**: There has been a notion that low socio-economic status was associated with preterm labour. There has been no association reported between the woman’s occupation (p=0.823), level of education (p=0.330), marital status (p=0.133) and preterm labour according to Wagura et al (5).

**Antenatal coverage**: Whereas a Kenyan study didn’t show any correlation between antenatal coverage and occurrence of preterm labour (p=0.621) (5), Hidayat et al (11), reported that women who attended more antenatal visits were more likely not have preterm labour. Those who had less than four visits were 1.865 times more likely to have preterm labour as compared to those who had more than four visits.

**Prior history of preterm labour**: According Hidayat (11), women with prior preterm labour were three times more likely to have preterm labour as compared to those who didn’t have prior history (p-value 0.01). Prior history of PROM especially prolonged increased the chances of preterm birth by 3.4 folds(19).

**Antepartum haemorrhage**: Women with antepartum haemorrhage were 6.835 times more likely to have preterm labour as compared to women with no vaginal bleeding (11). Wagura et al (5) reported a 4 fold increase in preterm labour among women who had antepartum hemorrhage. Placental abruption and placenta praevia were the commonest causes of this antepartum hemorrhage.

**Infections**: There has been conflicting evidence in regards to the impact of maternal infections on the occurrence of preterm labour. Bacterial vagnosis has been implicated in preterm labour. It changes the vaginal PH to above 5, thereby increasing the risk of preterm labour by 3-folds(20). Urogenital infections have been reported to raise the risk of preterm labour by increasing production of cytokines and prostaglandins (21). Chronic hepatitis B infections and HIV have shown no correlation with onset of preterm labour (22, 23).

**Nutritional deficiencies**: Studies show contradicting evidence on the impact of vitamin D levels on preterm labour. According to Wagner et al (24), lower levels of serum Vitamin D levels of less than 20ng/mL were associated with preterm labour. This was further emphasized in a meta-analysis (25) that levels <20ng/mL were also associated with preterm labour. There is need for more research though to justify Vitamin D supplementation in pregnancy as an intervention to prevent preterm labour. Wagura et al (5)reported no associated between maternal malnutrition (assessed using the Mid Upper Arm circumference) and preterm labour. Other studies in Indonesia (26) Malawi and elsewhere have reported that maternal malnutrition was associated with preterm labour (27, 28).

**Obstetric factors**: Having a caesarean delivery according to Wagura et al (5) was associated with nearly a two times chance of preterm labour than having a vaginal delivery (OR 1.832). Twin pregnancy was associated with nearly a 4-fold increase in preterm labour as compared to singleton pregnancy (OR 3.753) (11). Multiple gestation and polyhydramnios were associated with over distension of the uterus, thereby leading to preterm labour. Pregnancy induced hypertension led to a 5-fold increase in preterm labour (OR 5.20) (11).

Other factors which could lead to preterm labour include cigarette smoking, collagen vascular diseases (Ehlers-Danlos syndrome), polyhydramnios, anemia, chronic steroid therapy, invasive procedures (amniocentesis), cervical procedures (cone biopsies, cervical cerclage), and fetal anomalies(29).

2.1.4 Etiology:

A number of histologic studies demonstrate that the point of membrane leakage at term exhibits an area of altered morphology with thickening of the connective tissue components of the membranes, along with thinning of the cytotrophoblast layer and decidua. There is disruption of the connections between amnion and chorion thereby leading to focal leakage. It is hypothesized that such occurrences as seen in the physiological processes during normal labour could be occurring even with preterm labour thereby leading to focal leakage of amniotic fluid. At a cellular level, these changes result from the release of phospholipases, eicosanoids (especially prostaglandin E2), cytokines, elastases, matrix metalloproteinases, and/or other proteases in response to a physiologic or pathologic stimulus. Although the downstream cellular changes may be similar in preterm labour, the inciting etiologies in preterm PROM are likely different from term PROM (29).

2.2 Diagnosis**:**

There are a number of causes of preterm labour. The causes could follow infectious agents like *Plasmodium falciparum* in Malaria, premature PROM, polyhydramnios, hypertensive disorders of pregnancy and many more.

A diagnosis of premature rupture of membranes as a cause of preterm labour is made after taking a thorough history, physical examination and investigations. A patient with PROM can report a history of a sudden gush of fluid from her vagina which may be continuous especially on straining. A detailed history which may include prior sexual intercourse, presence of contractions, fever, foul smelling liquor or reduced fetal movements, is vital in identifying possible causes or presence of any complications like chorioamnionitis. It’s mandatory that the gestation age is determined using either the First day of the Last normal menstrual period or from prior dating ultrasound scans as this is helpful in streamlining patient management.

The diagnosis can further be confirmed on speculum examination, where there is pooling of fluid in the posterior fornix on coughing or on application of fundal pressure. Whereas it’s more appealing to the attending physicians to do a digital examination to avoid misdiagnosing an advanced preterm labour, that at times might delay timely referral, studies have shown that it reduces the latency period by nine days (30). Digital examination has also been associated with chorioamnionitis and neonatal sepsis (31). There is also evidence that visual assessment on speculum examination did not have any clinically significant difference from digital examination in determining advanced labour (32). On speculum examination, the attending physician should assess for presence or absence of the umbilical cord. In addition to determining the fetal presentation on an obstetric examination, the physician should assess for fetal reassurance. When a significant leakage of liquor has occurred, the fundal height might be smaller than expected.

Preterm labour can also be elicited by maternal infections. A detailed history of any fevers, foul smelling liquor, and abdominal pains is required. Women who have had prior cone biopsies for cervical dysplasia could also be at risk of cervical incompetence hence preterm labour. Women with history of uterine fibroids, multiple gestation and polyhydramnios are at increased risk of preterm labour. This history should be elicited and confirmed on physical examination (11).

**Confirmatory tests**:

Both the Nitrazine test and fernig tests can be carried out to confirm the diagnosis of PROM. The sensitivity of both of these tests approaches 90% (33). The normal vaginal PH is between 4.5 and 6.0. The Nitrazine test turns blue whenever the PH is above 6.0. False positive tests can however be seen in presence of Bacterial vagnosis, contaminations from semen, and blood. On speculum examination, separate swabs can be taken from the posterior fornix and vaginal walls and put on a slide. When it’s amniotic fluid, it arborizes and a typical fern distribution can be seen under a low power microscope. Other cervical swabs can be collected and sent for culture for Neisseria gonorrhea and chlamydia, whose presence is a significant risk factor for preterm labour. After removing the speculum, vaginal and perianal swabs can be collected and sent for group B streptococcus culture.

In cases where the patient’s history is suggestive of preterm labour but physical examination is inconclusive, ultrasound evaluation is so helpful. In addition to determining the liquor pool, ultrasonography helps in gestation age and fetal weight estimation, in determination of the placentation, presence of fetal anomalies, malpresentation and the number of fetuses. It can also be used in amniocentesis in determining lung maturation. To confirm leakage of amniotic fluid in PROM, 1ml of Indigo camine diluted with 9mls of sterile saline is instilled under ultrasound guidance. After 30 minutes, the vaginal swab will soiled to confirm the diagnosis. Methylene blue dye has been associated with hyperbilirubinemia and hemolytic anaemia in the infants (34), therefore it’s not advisable to use it for this purpose.

Several markers have been studied, including alpha fetoprotein (AFP),fetal fibronectin (fFN), insulin like growth factor binding protein 1 (IGFBP-1), prolactin, beta-subunit of human chorionic gonadotropin (hCG), creatinine, urea, lactate and placental alpha microglobulin 1 (PAMG-1) but the findings are still variable (29, 35).

2.3 Management:

The management of preterm labour majorly depends on the gestation age, non-reassuring fetal heart, presence of chorioamnionitis, or fetal malpresentation.

**2.3.1 Mode of delivery**:

The ideal mode of delivery in preterm labour especially with PROM is challenging in low resource settings (36-38) especially for gestation age remote from term. Women with preterm labour are delivered by caesarean section for indications such as prematurity, severe oligohydramnios in PPROM, malpresentation, or when the labour occurs in the presence of a previous caesarean scar. A significant number of these women however deliver vaginally before accessing the operating rooms (31). There are now some studies that show that vaginal delivery is as safe or even safer than caesarean delivery even in low resource settings (31). The available options of management include expectant or conservative management (especially for gestation age remote from term) or when lung maturity is not assured in absence of infections. The other options include augmentation of labour with either prostaglandins or oxytocin, and caesarean delivery.

**2.3.2 Medications:**

**Corticosteroids**:

There is overwhelming evidence that for gestation age less than 32 weeks, corticosteroids have shown reduced perinatal morbidity and mortality. Corticosteroid use has been associated with reduced intraventricular hemorrhage, necrotizing enterocolitis, respiratory distress syndrome (39). The recommended drugs and dosages are as follows; intramuscular Dexamethasone 6mg 12 hourly for 48 hours or intramuscular Betamethasone 12mg every 24 hours for 48 hours (40). There is no added benefit of giving more than four doses according to National Institutes of Health. Corticosteroid use after 34 weeks has shown to reduce the baby’s head circumference, length and body weight (41).

**Antibiotic use**:

Antibiotic use in preterm labour especially in PROM has been associated with reduced neonatal sepsis, neonatal pneumonia, postpartum endometritis, chorioamnionitis, and intraventricular hemorrhage. Antibiotic use has also been shown to increase the latency period (31, 42, 43). Penicillins are highly recommended either as a single drug or when combined. Following the National Institute of Child Health and Human Development trial, a combination of intravenous Ampicillin 2g and 250mg of Erythromycin250mg 6 hourly for 48 hours is highly recommended. This is followed by oral Amoxicillin 250mg 8 hourly and Erythromycin 250mg 6 hourly for 5-7 days (44). Other drugs have been studied including Azithromycin, Amoxicillin with clavulinic acid. When culture results show group B streptococcus, parenteral Penicillins should administered.

**Tocolysis**:

The role of Tocolysis in improving fetal outcomes is still controversial(45). Tocolysis may however cause short term prolonged latency period to allow transportation of the mothers to centers with better neonatal services, or to administer corticosteroids and the antibiotics. Magnesium sulphate as a tocolytic agent is thought to reduce the incidence of cerebral palsy before 32 weeks but more studies need to be conducted to justify its use in Preterm labour (46). It has also been shown to improve long-term neonatal morbidity and mortality (29). Long-term use of Tocolysis is however not recommended as there is no research to back up this practice.

**Other drugs used**: Different drugs can be used based on the different etiologies. Antihypertensive drugs can be in hypertensive disorders of pregnancy, Antimalarial drugs like Artesunate and Quinine can be used according to the local management protocols when malaria is confirmed in pregnancy.

2.4.1 Management of Preterm labour between 24 to 28 weeks:

In a number of African countries, viability has been set at 28 weeks. Preterm labour before 28 weeks is therefore termed as inevitable abortion. In developed countries, viability has been set at 24 weeks due to advanced neonatal care. Despite this difference in the health care systems, delivery before 32 weeks is associated with adverse perinatal outcomes. In absence of infection, it’s advisable to prolong latency period up to 34 weeks or until lung maturity has been confirmed (conservative management) by doing amniocentesis. Corticosteroids and antibiotics should be administered to improve perinatal outcomes. It should however be emphasized that the risk of chorioamnionitis and neonatal sepsis might increase with the prolongation of the pregnancy. Tocolysis can be administered to improve the perinatal morbidity like cerebral palsy and also buy more time to ensure administration of the antibiotics and corticosteroids. More research is needed to justify prolonged administration of tocolytic agents. Vaginal delivery is not contraindicated in patients with preterm labour at this gestation. Patients and their attendants should be informed that despite all the available interventions, a significant number of patients with PPROM still go into labour within one week(47). The contraindications to conservative management include presence of chorioamnionitis, confirmation of labour, non-reassuring fetal heart, cord prolapse, and placental abruption. Daily fetal monitoring is required which can be by obstetric assessment or by ultrasound scan. The obstetric team needs to consult with the neonatal team to improve on the perinatal outcomes. Digital examination in PROM is contraindicated. Measures to address the underlying causes of preterm labour like maternal infections are needed. Women with cervical incompetence can have cervical cerclage in the subsequent pregnancies.

2.4.2 Below 24 weeks:

Majority of patients with pre-viable PROM deliver within one week (48). Preterm labour before 24 weeks is associated with adverse perinatal outcomes which include motor and neurodevelopment disorders like cerebral palsy, chronic lung diseases, potter syndrome, and lung hypoplasia. It’s therefore advisable that the patients are informed of these possible outcomes as the pregnancy might not continue to reach gestation ages leading to normal infants. The obstetric teams need to make consultations with the perinatologists or neonatologists to improve these outcomes. The care team should assess for fetal wellbeing daily. The team should also be on the lookout for signs of maternal infections like maternal tachycardia, fevers, uterine tenderness, foul smelling liquor. Management of patients with preterm labour before 24 weeks is controversial. Bed rest and sexual abstinence is however advisable.

3.0 METHODS:

3.1 Study Design**:** Descriptive hospital based Cross-sectional Study

Women admitted with preterm labour between 24 and 28 weeks of gestation who consent to participate in the study will be followed from admission to discharge from hospital. The status of their babies will be assessed; admission to special care unit and their ultimate status at discharge from the special care unit will be noted.

3.2 Study Setting**:**

This study will be conducted in the obstetric wards of Kawempe General Hospital. Kawempe General Hospital is located about 12 kilometers (7.5 miles) from Kampala City Center, by road, north of the city's central business district, along the Kampala-Gulu highway. It’s located in Kawempe Division. It is one of the teaching hospitals for Makerere University College of Health Sciences (MakCHS). Kawempe General Hospital is a government-funded hospital with a bed capacity of about 900, although it always exceeds this capacity. It serves a population of approximately 4.5 million.

The study units will include the labour suite, postnatal ward, labour suite operating theatre, and HDU (High Dependency Unit) and the Neonatal Special Care Unit. The units operate 24 hours per day and offer free services to the public. Patients come from within Kampala, as well as neighboring and distant districts.

3.3 Study Period**:** One year

3.4 Case definition of study participants**:**

The criteria for diagnosing study participants will follow a history of labour like pains between 24 and 28 weeks of gestation. Upon consenting to participate in the study, a detailed patient history will be obtained. This will include ascertaining presence of labour pains, show, drainage of liquor, evidenced by cervical effacement and dilation on physical examination to confirm the diagnosis of labour. When premature rupture of membranes is suspected, a sterile speculum examination will be undertaken to exclude PROM. An obstetric ultrasound will be undertaken to determine viability, gestation age, and any obvious fetal anomaly. The pregnancies will be dated using the first day of the last menstrual period or using obstetric ultrasound scans whenever accessible.

3.5 Inclusion criteria:

Any woman admitted with evidence of preterm labour with a single or multiple viable fetuses between 24 and 28 weeks of gestation who consents to participate in the study.

3.6 Exclusion Criteria:

Women with preterm labour between 24 and 28 weeks of gestation with fetal demise or with congenital anomalies or not sure of dates will be excluded. Women diagnosed and managed for preterm labour in other facilities but referred after delivery will also be excluded.

**3.7 Sampling procedure**:

Mother-baby pairs who meet the inclusion criteria will be recruited upon giving informed consent using systematic sampling from the labour and emergency gynaecology wards to the postnatal wards and followed up to discharge from hospital. A pretested standard questionnaire will be administered to the mothers with preterm labour between 24 and 28 weeks. Baby and mothers’ medical records will also be used when additional information is needed for clarity.

3.8 Sample Size Calculation**:**

Using OpenEpi sample size calculator, a review of Kawempe General Hospital records from January 2019 to March 2019, shows that patients with preterm labour were 74 out 326 abortions. This would translate into a percentage of those with outcome of interest being 0.227, we calculated a sample size needed of 348 after computing into the OpenEpi, using a power of 80% and significance at p<0.05. See figure below


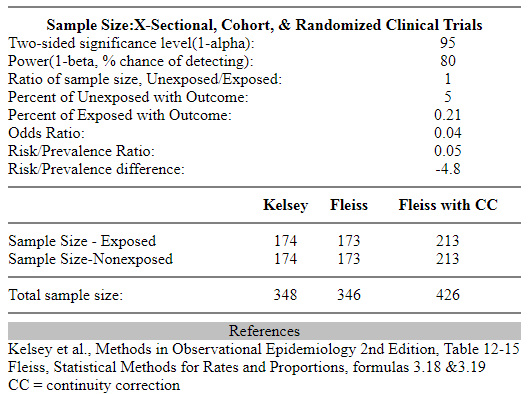


Taking into account of a 10% loss to follow up, we shall recruit 383 participants into the study.

3.9 Outcome variables:

3.9.1 Primary Outcome: Preterm birth

3.9.2 Neonatal Variables: Admission to special care unit (SCU), Neonatal mortality, respiratory distress syndrome, neonatal infections (defined by evidence of high grade fever, failure to feed, vomiting or convulsions), neonatal convulsion, APGAR score at delivery, and need for neonatal resuscitation.

3.9.3 Maternal Variables: Parity, age, antenatal care attendance, duration of hospital stay, mode of delivery, evidence of chorioamnionitis defined by fever and clinical exam consistent with endometritis, initiation of antibiotics, postpartum hemorrhage, wound sepsis, wound separation, HIV sero-status and maternal death.

3.10 DATA MANAGEMENT**:**

310.1 Data collection procedure**:**

Using a data collection tool, information on each study participant will be collected. This will include: their bio-data, the investigations undertaken, the management given (Antibiotics, corticosteroids, tocolytics), Mode of delivery, maternal and neonatal outcomes. A pilot study will be conducted on 20 participants to pretest and improve the data collection tools. Upon consenting, history and physical examination will be carried out on all study participants by trained research assistants. An obstetric ultrasound scan will be carried out to confirm viability, presentation, liquor pool and any obvious fetal anomalies. According to the planned mode of delivery by the obstetric team on duty, the participants will be followed prospectively from admission up to discharge from the hospital. The study will run over 24 hours, Monday up to Saturday every week. Data collected will be crosschecked and entered on the same day of collection.

3.10.2 Statistical analysis**:**

Data entry will be done using EPI-DATA 3.1 and analyzed using STATA version 14. Baseline characteristics will be described using descriptive statistics presented in frequencies, means and percentages as appropriate. We shall determine the prevalence of preterm labour by determining the percentage of women with preterm labour between 24 and 28 weeks of gestation out of the total deliveries during the study period per 1000 deliveries. Inferential statistics will be used to establish the association between preterm birth and various risk factors using the Chi-square analysis. Outcome variables that will be associated with preterm births with p-value 0.2 in bivariate analysis will be entered into a multivariate logistic model with preterm birth and mode of delivery. Using multilevel logistic regression, we shall determine the risk factors independently associated with preterm birth. Interaction among variables in the multivariate model will be checked.

3.10.3 Quality Control**:**

Four Research Assistants will be trained prior to the data collection. Two research assistants will collect data during day and the other two at night. A pilot study will be carried out to pretest and modify the data collection tools. Data collection tools will be checked for completeness. Thereafter it will be edited, coded and entered on same day of collection. Data will be backed up and kept under limited access.

3.11 Ethical Consideration**:**

Permission to conduct the study will be sought from;

- Directorate of Obstetrics and Gynecology Makerere University
- Ethics Review Board
- Informed Consent from the study Participants
- Uganda National Council of Science and Technology

3.12 Study Limitations**:**

- Poor record management may hinder access to some participants’ files. This will be partly overcome by having trained research assistants working in a 24-hour rotation to keep track of the participants’ files.
- Inadequate recording in participants’ files by the medical teams may limit collection of vital data. The may alter the findings of the study. Participants’ will be asked whether particular interventions were given even when not well recorded in their files.
- Change of a given mode of delivery by the medical teams than earlier planned may bias the outcome of one mode of delivery over the other.
- Inability to control health Systems factors like shortage of supplies or limited human resource.
- Confounding factors like level of health workers’ expertise may override the others’ planned mode of delivery.

### 3.13 Project Timeline and Plan

My goal is to complete the study within one year. I plan to dedicate the last month to writing and submission for presentation and publication. Given the volume of the maternity ward and my familiarity with the people and clinical setting of Kawempe General Hospital, I believe this is a feasible goal. I plan to use the skill set and knowledge that I acquire from this funding opportunity primarily in research to conduct future studies and to begin to apply for larger competitive funding. I believe that with more skill acquisition, I’ll be a better mentor for both undergraduate and postgraduate students.

4.0 REFERENCES:

1. Edwin Chandraharan S. Recent advances in management of preterm labor. J Obstet Gynecol India. 2005;55(2):118-24.

2. Slattery MM, Morrison JJ. Preterm delivery. The Lancet. 2002;360(9344):1489-97.

3. Bhutta AT, Cleves MA, Casey PH, Cradock MM, Anand K. Cognitive and behavioral outcomes of school-aged children who were born preterm: a meta-analysis. Jama. 2002;288(6):728-37.

4. Blencowe H, Cousens S, Oestergaard MZ, Chou D, Moller A-B, Narwal R, et al. National, regional, and worldwide estimates of preterm birth rates in the year 2010 with time trends since 1990 for selected countries: a systematic analysis and implications. The lancet. 2012;379(9832):2162-72.

5. Wagura P, Wasunna A, Laving A, Wamalwa D. Prevalence and factors associated with preterm birth at kenyatta national hospital. BMC pregnancy and childbirth. 2018;18(1):107.

6. World Health Organization. World health statistics 2015:

7. Otieno P, Waiswa P, Butrick E, Namazzi G, Achola K, Santos N, et al. Strengthening intrapartum and immediate newborn care to reduce morbidity and mortality of preterm infants born in health facilities in Migori County, Kenya and Busoga Region, Uganda: a study protocol for a randomized controlled trial. Trials. 2018;19(1):313.

8. Uganda MoH. Ministry of Health Commemorates World Prematurity Day 2018. Kampala: 2018.

9. department MHR. Annual report for Department of Obstetrics and Gynecology. 2017/18.

10. Lawn JE, Kinney M. Preterm birth: now the leading cause of child death worldwide. American Association for the Advancement of Science; 2014.

11. Hidayat ZZ, Ajiz EA, Krisnadi SR. Risk factors associated with preterm birth at hasan sadikin general hospital in 2015. Open Journal of Obstetrics and Gynecology. 2016;6(13):798.

12. Betrán AP, Torloni MR, Zhang J-J, Gülmezoglu A, Section WWGoC, Aleem H, et al. WHO statement on caesarean section rates. BJOG: An International Journal of Obstetrics & Gynaecology. 2016;123(5):667-70.

13. Stewart CJ, Tregoning SK, Moller G, Wainwright H. Preterm prelabour rupture of the membranes before 28 weeks: better than feared outcome of expectant management in Africa. European Journal of Obstetrics & Gynecology and Reproductive Biology. 2006;126(2):186-92.

14. Morales WJ, Talley T. Premature rupture of membranes at< 25 weeks: a management dilemma. American journal of obstetrics and gynecology. 1993;168(2):503-7.

15. Goldenberg RL, Culhane JF, Iams JD, Romero R. Epidemiology and causes of preterm birth. The lancet. 2008;371(9606):75-84.

16. Born Too Soon. The Global Action Report on Preterm Birth. Geneva: World Health Organization. 2012.

17. Savitz DA, Blackmore CA, Thorp JM. Epidemiologic characteristics of preterm delivery: etiologic heterogeneity. American journal of obstetrics and gynecology. 1991;164(2):467-71.

18. Kozuki N, Lee AC, Silveira MF, Sania A, Vogel JP, Adair L, et al. The associations of parity and maternal age with small-for-gestational-age, preterm, and neonatal and infant mortality: a meta-analysis. BMC public health. 2013;13(3):S2.

19. Di Renzo GC, Giardina I, Rosati A, Clerici G, Torricelli M, Petraglia F, et al. Maternal risk factors for preterm birth: a country-based population analysis. European Journal of Obstetrics & Gynecology and Reproductive Biology. 2011;159(2):342-6.

20. Foroozanfard F, Tabasi Z, Mesdaghinia E, Sehat M, Mehrdad M. Cervical length versus vaginal PH in the second trimester as preterm birth predictor. Pakistan journal of medical sciences. 2015;31(2):374.

21. Verma I, Avasthi K, Berry V. Urogenital Infections as a risk factor for preterm labor: A hospital-based case–control study. The Journal of Obstetrics and Gynecology of India. 2014;64(4):274-8.

22. Coley JL, Msamanga GI, Fawzi MCS, Kaaya S, Hertzmark E, Kapiga S, et al. The association between maternal HIV-1 infection and pregnancy outcomes in Dar es Salaam, Tanzania. British Journal of Obstetrics and Gynaecology. 2001;108(11):1125-33.

23. Huang Q-t, Wei S-s, Zhong M, Hang L-l, Xu Y-y, Cai G-x, et al. Chronic hepatitis B infection and risk of preterm labor: a meta-analysis of observational studies. Journal of Clinical Virology. 2014;61(1):3-8.

24. Wagner C, Baggerly C, McDonnell S, Baggerly L, Hamilton S, Winkler J, et al. Post-hoc comparison of vitamin D status at three timepoints during pregnancy demonstrates lower risk of preterm birth with higher vitamin D closer to delivery. The Journal of steroid biochemistry and molecular biology. 2015;148:256-60.

25. Qin L-L, Lu F-G, Yang S-H, Xu H-L, Luo B-A. Does maternal vitamin D deficiency increase the risk of preterm birth: a meta-analysis of observational studies. Nutrients. 2016;8(5):301.

26. Kalanda B. Maternal anthropometry and weight gain as risk factors for poor pregnancy outcomes in a rural area of southern Malawi. Malawi medical journal: the journal of Medical Association of Malawi. 2007;19(4):149.

27. Sebayang SK, Dibley MJ, Kelly PJ, Shankar AV, Shankar AH, Group SS. Determinants of low birthweight, small‐for‐gestational‐age and preterm birth in Lombok, Indonesia: analyses of the birthweight cohort of the SUMMIT trial. Tropical Medicine & International Health. 2012;17(8):938-50.

28. Han Z, Mulla S, Beyene J, Liao G, McDonald SD. Maternal underweight and the risk of preterm birth and low birth weight: a systematic review and meta-analyses. International journal of epidemiology. 2010;40(1):65-101.

29. Caughey AB, Robinson JN, Norwitz ER. Contemporary diagnosis and management of preterm premature rupture of membranes. Reviews in obstetrics and gynecology. 2008;1(1):11.

30. Lewis DF, Major CA, Towers CV, Asrat T, Harding JA, Garite TJ. Effects of digital vaginal examinations on latency period in preterm premature rupture of membranes. Obstetrics and gynecology. 1992;80(4):630-4.

31. Kayiga H, Lester F, Amuge PM, Byamugisha J, Autry AM. Impact of mode of delivery on pregnancy outcomes in women with premature rupture of membranes after 28 weeks of gestation in a low-resource setting: A prospective cohort study. PloS one. 2018;13(1):e0190388.

32. Munson LA, Graham A, Koos BJ, Valenzuela GJ. Is there a need for digital examination in patients with spontaneous rupture of the membranes? American journal of obstetrics and gynecology. 1985;153(5):562-3.

33. Buyukbayrak E, Turan C, Unal O, Dansuk R, Cengizoğlu B. Diagnostic power of the vaginal washing-fluid prolactin assay as an alternative method for the diagnosis of premature rupture of membranes. The Journal of Maternal-Fetal & Neonatal Medicine. 2004;15(2):120-5.

34. Naylor CS, Gregory K, Hobel C. Premature rupture of the membranes: an evidence-based approach to clinical care. American journal of perinatology. 2001;18(07):397-414.

35. Gaucherand P, Guibaud S, Awada A, Rudigoz RC. Comparative study of three amniotic fluid markers in premature rupture of membranes: fetal fibronectin, alpha-fetoprotein, diamino-oxydase. Acta obstetricia et gynecologica Scandinavica. 1995;74(2):118-21.

36. Chakraborty B, Mandal T, Chakraborty S. Outcome of Prelabor Rupture of Membranes in a Tertiary Care Center in West Bengal. 2013.

37. Mousiolis A, Papantoniou N, Mesogitis S, Baglatzi L, Baroutis G, Antsaklis A. Optimum mode of delivery in gestations complicated by preterm premature rupture of the membranes. The Journal of Maternal-Fetal & Neonatal Medicine. 2012;25(7):1044-9.

38. Eleje G, Ezebialu I, Umeobika J, Eke A, Ezeama C, Okechukwu Z. Pre-labour rupture of membranes at term: a review of management in a health care institution. Afrimedic Journal. 2010;1(2):10-4.

39. Harding JE, Pang J-M, Knight DB, Liggins GC. Do antenatal corticosteroids help in the setting of preterm rupture of membranes? American Journal of Obstetrics & Gynecology. 2001;184(2):131-9.

40. Gilstrap LC, Christensen R, Clewell WH, D'Alton ME, Davidson EC, Escobedo MB, et al. Effect of corticosteroids for fetal maturation on perinatal outcomes: NIH consensus development panel on the effect of corticosteroids for fetal maturation on perinatal outcomes. Jama. 1995;273(5):413-8.

41. Vidaeff AC, Doyle NM, Gilstrap III LC. Antenatal corticosteroids for fetal maturation in women at risk for preterm delivery. Clinics in perinatology. 2003;30(4):825-40.

42. Medina TM, Hill DA. Preterm premature rupture of membranes: diagnosis and management. Am Fam Physician. 2006;73(4):659-64.

43. Mercer BM, Arheart KL. Antimicrobial therapy in expectant management of preterm premature rupture of the membranes. The Lancet. 1995;346(8985):1271-9.

44. Kenyon S, Boulvain M, Neilson J. Antibiotics for preterm rupture of membranes. Cochrane Database Syst Rev. 2003;2(2).

45. Weiner CP, Renk K, Klugman M. The therapeutic efficacy and cost-effectiveness of aggressive tocolysis for premature labor associated with premature rupture of the membranes. American Journal of Obstetrics & Gynecology. 1988;159(1):216-22.

46. Di Renzo GC, Roura LC, Facchinetti F, Antsaklis A, Breborowicz G, Gratacos E, et al. Guidelines for the management of spontaneous preterm labor: identification of spontaneous preterm labor, diagnosis of preterm premature rupture of membranes, and preventive tools for preterm birth. The Journal of Maternal-Fetal & Neonatal Medicine. 2011;24(5):659-67.

47. Schucker JL, Mercer BM, editors. Midtrimester premature rupture of the membranes. Seminars in perinatology; 1996: Elsevier.

48. Schutte M, Treffers P, Kloosterman G, Soepatmi S. Management of premature rupture of membranes: the risk of vaginal examination to the infant. American Journal of Obstetrics & Gynecology. 1983;146(4):395-400.

# 5.0 WORK PLAN:

|  | June  2019 | July  2019 | Aug  2019 | Sept  2019 | Oct  2019 | Nov  2019 | Dec  2019 | Jan  2020 | Feb  2020 | Mar  2020 | April  2020 | May  2020 | June  2020 | July  2020 | Aug  2020 | Sept  2020 | Oct  2020 |
| --- | --- | --- | --- | --- | --- | --- | --- | --- | --- | --- | --- | --- | --- | --- | --- | --- | --- |
| Obs  Dept | X |  |  |  |  |  |  |  |  |  |  |  |  |  |  |  |  |
| TASO  Ethics  Board |  | X | X |  |  |  |  |  |  |  |  |  |  |  |  |  |  |
| Uganda  Regist |  |  |  |  | X |  |  |  |  |  |  |  |  |  |  |  |  |
| Pretest  Data  Coll  Tools |  |  |  |  | X | X |  |  |  |  |  |  |  |  |  |  |  |
| Data  Coll. |  |  |  |  |  |  | X | X | X | X | X | X | X | X |  |  |  |
| Data  analysis |  |  |  |  |  |  |  |  |  |  |  |  |  |  | X | X |  |
| Writing  Manusc  Prep.  Present |  |  |  |  |  |  |  |  |  |  |  |  |  |  | X | X | X |

# 6.0 BUDGET:

|  | Amount US Dollars |
| --- | --- |
| Research Assistant x 4 for 24/7 staffing | 200 x 4 = 800 |
| Data Entry | 300 |
| Records Personnel | 400 |
| Photocopying, supplies, computer, software | 300 |
| IRB | 300 |
| UNSCT | 300 |
| Data analysis | 300 |
| Manuscript preparation and writing | 300 |
| Participants’ fees | 1,000 |
| **Total** | 4,000 |

APPENDIX I: MENTORSHIP PLAN

**Josaphat Byamugisha, Associate Professor, Department of Obstetrics and Gynecology**

Dr. Byamugisha’s numerous qualifications include a PhD in Reproductive Health. He has over 20 years of experience in clinical and reproductive health research. He is currently the PI on a NIH R21 grant on fistula reintegration. His past research projects include grants from PATH, PSI Uganda as well as collaborations with UCSF, Harvard and Case Western. He has over 20 peer review publications. Initially I will meet with Dr. Byamugisha weekly throughout the IRB process. We will then meet monthly to discuss progress and he will also be involved in monthly Skype sessions. In addition to guidance with the IRB and further refinement of the project methodology, Dr. Byamugisha will help me navigate the complexities of the clinical and medical records aspects of Mulago hospital. He will be integral in manuscript preparation and editing. He will also be my primary career advisor for continuing a career in academic medicine in my home country.

**Dr. Annette Nakimuli, Assoc. Professor &Chair, Department of Obstetrics &Gynaecology**

Dr. Annette Nakimuli is an Associate Professor of Obstetrics and Gynecology in Makerere University College of Health Sciences. She holds a PhD, a Master’s degree in Obstetrics and Gynecology from Makerere University, a Bachelor of Medicine and Bachelor of Surgery degree from Makerere University. Dr. Nakimuli has extensive clinical research experience and has over 20 publications in international peer-reviewed journals.

Initially I will meet with Dr. Nakimuli weekly throughout the IRB process and she will provide statistical guidance throughout the project. We will then meet monthly to discuss progress and she will also participate in the monthly Skype sessions. She will be particularly involved in methodology, analysis and manuscript production.

# APPENDIX II: CONSENT

**INFORMATION FOR THE STUDY PARTICIPANTS:**

**Introduction:** I am Dr. Herbert Kayiga, a practicing Obstetrician/Gynaecologist, also a Lecturer in the department of Obstetrics and Gynaecology, Makerere University College of Health Sciences, a researcher in the study “*Prevalence, Risk factors and ideal Mode of delivery following Preterm labour between 24 to 28 weeks of gestation in a low resource setting”*. This study will be conducted in Kawempe General Hospital found in Kampala. It is funded by the HEPI project under Makerere University College of Health Sciences.

I am here to request you to participate in the study. Before you decide let me give you some information about the study. If you agree to take part, you will be required to sign consent form.

**Purpose of the study:** In this study, we are going to interview you on the likely causes of your going into labour before the ideal expected time of delivery and the route through which deliver your baby or babies. This will be from the time you got signs and symptoms of premature labour between 24 and 28 weeks, throughout your stay in hospital to the point you leave the hospital. This will help us improve the way we treat you or other patients with the same condition as you. The condition you makes you to go into labour before the ideal expected time of delivery. Approximated time for this interview is 30 minutes.

**Study Procedure:** You will be interviewed about your condition (preterm labour), when it started, what has been done so far, likely mode of delivery, the length of your stay in hospital and the outcome your baby or babies at discharge. We’ll use the information you give us to clarify some gaps in your case file. We aim to interview 383 patients including you.

**Risks:** Minimal risk.

**Rights:** You have a right to decline to participate in the study or withdraw from it at any stage of questioning without affecting the quality of care offered to you while you are in hospital.

**Confidentiality:** All your responses will be kept confidential. Your identity will not be revealed to the staff attending to you unless you give consent or assent.

**Benefits:** There are no direct benefits to you but the information gathered will help us to improve the quality of care we offer to patients diagnosed and managed for preterm labour between 24 and 28 weeks.

**Cost and Compensation**: You will be given 20,000/= (Twenty thousand shillings) as a compensation for your time to participate in the study.

**Feedback to participants:** You will be given feedback on the progress or the findings of the study.

**Questions:** If there are any questions you would like to ask you can do so now or contact the following the Principal Investigator.

The contacts are:

1. **Dr. Herbert Kayiga**

Directorate of Obstetrics and Gynecology,

Makerere University College of Health Sciences

0777855063.

Email: [hkayiga@gmail.com](mailto:hkayiga@gmail.com)

**2. Dr. Etukoit Bernard**

Chairperson: TASO IRB/REC (Research & Ethics Committee)

TASO Uganda

+256414532580/1

Email: [etukoitm@tasouganda.org](mailto:etukoitm@tasouganda.org)

1. **Dr. Peter Ndemere**

Executive Secretary- UNCST

+256414705500

Email: [info@uncst.go.ug](mailto:info@uncst.go.ug)

# APPENDIX III: CONSENT FORM

**STATEMENT OF CONSENT**

I the undersigned acknowledge that the principal investigator/the research assistant has fully explained to me the nature, purpose and procedures involved in this study. I appreciate that participation is completely voluntary; that my refusal or withdrawal from this study will not in any way affect any medical service or medical advice I may need now and in the future.

I therefore sign here as proof of my consent for participating in this study.

Name……………………………………………………………………………………

Signature or Right thumb imprint………………………………………………………

Date……………………………………………………………………………………

I have explained to the best of my knowledge the purpose of this study to the participant and her consent has been without force or coercion. I have given the participant enough time to understand what the study is about in a language; she is well vested with. I also acknowledge that I have given her an opportunity to ask questions for clarity.

Name of person obtaining consent……………………………………………………

Signature………………………………………………………………………………

Date……………………………………………………………………………………

# APPENDIX IV: LUGANDA CONSENT

**Title:** **MBEERA KI ERETERA ABAKYALA OKUZAALA WAKATI WE MYEZI OMUKAAGA N’OMUSANVU, ERA NGERIKI EY’OKUZAALA EYINZA OKUKENDEEZA OKUFFA KW’ABAANA SINGA OSANGIBWA NGA OLUMWA: TUTUNULIRA OKUSINDIKA OMWAANA OBA OKULONGOSEBWA MU DDWALIRO EKKULU E KAWEMPE.**

**Ennyanjula:**

Nze (Errinya) anoonyereza ku ngeriki ey’okuzaala eyinza okukendeza okuffa kw’abaana ssinga osangibwa nga olumwa wakati w’emyezi omukaaga n’omusanvu. Tutunulira okusindika omwaana oba okulongosebwa mu ddwaliro ekkulu e Kawempe.

Nkusaba /muwalawo/ow’oluganda wo (Londako) okwetaba mukunoonyereza kuno. Nga tonasalawo kankubulire ebikwata ku kunoonyereza kuno. Bw’onokiriza okwetaba mu kunoonyereza kuno ojja kusabibwa okusayininga oba okussa ekinkumu kyo ku kiwandiiko kino.

**Omulamwa n’ekigendererwa mu kunoonyereza kuno:**

Mukunoonyereza kuno, tujja kubuuza ebikwata kubulamu bwo engeri gy’ojjanjabidwamu n’emitendera gy’oyisemu nga oli mu ddwaliro okusinziira kumbeera gyewalimu. Kino kijja kutuyamba okulongoosa embeera y’okukujjanjaba wamu n’abalwadde abalala abali mu mbeera nga eyiyo. Embeera gy’olimu eretera akasuwa k’amazzi agali ku’mwana okwabika. Wesanga ng’ otobye mungoye ezomunda. Tujja kumala edakika nga 30 (asatu) nga twogeramu nawe oba nga tukubuuza ebikukwatako.

**Ebinagobererwa mu kunonyereza kuno**:

Tujja kubuuza ku bujjanjabi bwewaakafuna nga oli mu ddwaliro. Oluvanyuma lw’okukeberwa omusawo, bw’amanya nti olina enzaala y’okusikatira, tujja kweyambisa by’onotubulira ebikukwatako twetegereze oba twekanye ebyo ebiyinza okuba nga byebikuviirako okubeera mu mbeera bwetyo.Tugenda kwebuuza ku balwadde 383 (bisatu kinaana mu basatu) nga nawe mwoli.

**Obuzibu oba obulabe mu kunoonyereza kuno:** Tewali buzibu oba bulabe bwamanyi buli mukunoonyereza kuno.

**Eddembe lyo mu kunoonyereza kuno:**

Olina eddembe okugaana okwetaba mu kunoonyereza kuno oba okuvaamu ekiseera kyona awatali kujjako oba kutabula bujjanjabi bwolina kufuna mu ddwaliro lino.

**Okukuuma ebyaama mu kunoonyereza kuno:**

Ebikukwatako byona byakukumibwa bulungi nga byakyaama era erinnya lyo ssi lya kuweebwa oba kulagibwa abasawo abakukolako okujako nga bamazze okufuna olukusa okuva ewuwo.

**Eby’okuganyulwa mukunonyereza:**

Tojja kuganyulwa butelevu mukunoonyereza kuno wabula ebinazuulibwa bijja kutuyamba mukulongoosa enzijjanjaba eri abalwadde abazuulidwa nga balumwa wakati w’emyezi omukaaga n’omusanvu.

**Ebyensasula mu kunoonyereza kuno:** Ojja kusasulwa 20,000 Ugx okwetaba mu kunoonyereza kuno.

**Ebibuuzo:**

Bwoba n’ebibuuzo byona ebikwata ku kunoonyereza kuno ekiseera kyona, tukilira bano wamanga;

**1. Omusawo (Dr.) Herbert Kayiga**

Directorate of Obstetrics and Gynecology,

Makerere University College of Health Sciences.

0777855063.

Email: [hkayiga@gmail.com](mailto:hkayiga@gmail.com)

1. **Kalabalaba w’okunonyereza kuno**

**Dr. Etukoit Bernard**

Chairperson: TASO IRB/REC (Research & Ethics Committee)

TASO Uganda

+256414532580/1

Email: [etukoitm@tasouganda.org](mailto:etukoitm@tasouganda.org)

1. **Dr. Peter Ndemere**

Executive Secretary- UNCST

+256414705500

Email: info@uncst.go.ug

**OKUKIRIZA OKWETABA MUKUNOONYEREZA**

Nkakasa nti akulira okunoonyereza kuno oba amuyambako ambulidde/atubulidde bulungi ebigendelerwa n’ebinagobererwa mukunoonyereza kuno. Manyi nti okwetabamu kwange kwa kyeyagalire era okugaana okukwetabamu oba okuvaamu tekigenda kutabula bujjanjabi bwe nina kufuna kati oba ne mubiseera by’omumaaso.

Omukono oba ekinkumu kyange wamanga kiraga nti nzikiriza kyeyagalire.

Erinnya………………………………………………………………………………..

Omukono oba ekinkumu……………………………………………………………...

Ennaku z’omwezi………………………………………………………………………

Mbulidde bulungi ebigendelerwa by’okunoonyereza kuno eri ono akwetabyemu era akirizza kyeyagalire. Muwadde obudde okwetegereza n’okusoma mu kiwanddiko mu lulimi lw’ategeera era nkakasa nti muwadde omukisa okubuuza ebibuuzo by’atategedde.

Amannya…………………………………………………………………………………

Omukono/ekinkumu ……………………………………………………………………

Ennaku z’omwezi………………………………………………………………………

………………………

#

# APPENDIX V: DATA COLLECTION TOOL

**Part A: SECTION 1: SOCIO- DEMOGRAPHIC DATA**

| **Question**  **Number** | **Questions** | **Response categories** |  | **Skip to** |
| --- | --- | --- | --- | --- |
| **1.** | Date of case recruitment | …………………………………... |  |  |
| **2.** | Register/Unit No. | Number..........................................  …………………………………... |  |  |
| **3.** | Patient’s Age | ………………………………… |  |  |
| **4.** | Gravidity | ……………………………….. |  |  |
| **5.** | Occupation | 1. Employment……………1 2. House wife/unemployed..2 |  | **If 2 skip to 7** |
| **6.** | How much do you earn monthly in shillings? | <50,000/=……………………….…1  50,000-<500,000/=………………..2  500,000-<1,000,000/=…………….3  >1,000,000/=……………………...4 |  |  |
| **7.** | HIV status | Positive ……………………………1  Negative……………………..........2 |  |  |
| **8.** | Smoking | Does smoke………………………1  Does not smoke…………………..2 |  |  |
| **9.** | Prior caesarean scars | No prior scar……………………….1  1 scar……………………………....2  2 or scars…………………………...3 |  |  |
| **10.** | Prior history of drainage of liquor | Present……………………………..1  Absent……………………………..2 |  |  |
| **11.** | Outcome of previous pregnancy | Live Premature…………………….1  Live full term………………………2  FSB………………………………...3  MSB……………………………….4  ENND……………………………...5  Congenital anomalies……………...6  Multiple gestation………………….7 |  |  |
| **12.** | Have you ever had any operations on your cervix? | No……………….…………………1  Yes...……………………………….2 |  | **If No, skip to 14** |
| **13.** | Which cervical procedure did you have? | Cervical procedures for cancer of cervix (LEEP or biopsy),………..…1  Procedures to help me keep my baby in (cervical cerclage)………………2 |  |  |
|  | **Diagnosis** |  |  |  |
|  | **Clinical state on admission:** |  |  |  |
| **14.** | Did you have labour pains before admission? | No………………………………1  Yes…………………………..…2 |  |  |
| **15.** | Did you have fever prior to admission? | No…...………………………….1  Yes….………………………….2 |  | **If No, skip to 17** |
| **16.** | What is the definitive diagnosis by the care team? | Malaria…………………………1  UTI…………………………….2  Chorioamnionitis………………3  Other (specify)………………….4 |  |  |
| **17.** | Was there vaginal bleeding before the pain? | No……………………..………..1  Yes…………………………..….2 |  | **If No skip to 19** |
| **18.** | Did you get any of the following before the vaginal bleeding? | Did you fall or get involved in any accident?…………………..1  Were you assaulted before bleeding?………………………2  The vaginal bleeding started spontaneously………………….3 |  |  |
| **19.** | Was your labour induced by the medical team? | No………………………………1  Yes……………………………...2 |  | **If No, skip to 21** |
| **20.** | Were any of the following the reasons for the induction? | Hypertensive conditions in pregnancy………………………1  Diabetes mellitus……………….2  Sickle cell anaemia…………….3  My baby’s condition was in danger………………………….4  My baby had abnormalities……5  Other (specify)…………………6 |  |  |
| **21.** | Did you have drainage of liquor before labour pains? | No………………………...…….1  Yes………………………..……2 |  | **If No, skip to 27** |
| **22.** | Duration of drainage of liquor | <24 hours………………….……….1  24<48hours…………………...……2  2-7 days…..……..………...…….…3  >7days………………..……………4 |  |  |
| **23.** | Color of Liquor | Clear………………………….……1  Lightly stained meconium…….…...2  Thick stained meconium……….….3 |  |  |
| **24.** | Smell of liquor | Offensive smell…........……………1  Non offensive smell………….……2 |  |  |
| **25.** | Was speculum Examination done? | Yes……………………..…………..1  No………………………….………2 |  |  |
| **26.** | Number of digital examinations/day | 0……………………………………1  1-2………………………….………2  3-4………………………….………3  >5 ………………………………….4 |  |  |
| **27.** | Was the fetal Heart heard at admission? | No……..….…………….………….1  Yes…………………………………2 |  |  |
| **28.** | Was an Obstetric Ultrasound scan done? | No………………………………….1  Yes…………………………………2 |  | **If No, skip to 30** |
| **29.** | What were the U/s findings? | Viability……………………..……..1  Placentation…………………….….2  Liquor pool………………………...3  Number of babies………………….4  Presentation/lie…………………….5 |  |  |
| **30.** | How many babies are you carrying in the current pregnancy? | Singleton (one)……………………1  More than one baby………………..2  Not sure……………………………3 |  |  |
|  | **Delivery** |  |  |  |
| **31.** | What is the planned mode of management after diagnosis? | Expectant management……………1  Active management…………..……2 |  | **If expectant management skip to 33** |
| **32.** | What is the planned mode of delivery if active management is chosen? | Induction with Misoprostol….…….1  Induction with Pitocin……………..2  Augmentation………………….…..3  TOLAC………………………….....4  Caesarean delivery…………….…..5  Destructive delivery………….........6 |  |  |
| **33.** | What is actual mode of delivery? | Vaginal delivery………………..….1  Assisted Vaginal delivery………….2  Emergency caesarean……………...3  Destructive delivery…………….....4 |  |  |
| **34.** | What is duration from decision making to delivery? | < 24 hours………………………....1  1<2 days…..…………………….…2  2<3 days…..…………………….....3  3< 7 days…….………...………..…4  >7 days…………………………….5 |  |  |
|  | **Drugs** |  |  |  |
| **35.** | If < 34 weeks gestation, where corticosteroids given? | No………………………………….1  Yes…………………………………2 |  | **If No, skip to 37.** |
| **36.** | Which corticosteroids were given? | Dexamethasone……………………1  Betamethasone……………….…....2  Hydrocortisone………………….....3  Others (Specify)…………………...4 |  |  |
| **37.** | Were Antibiotics given? | No…………………………….……1  Yes…………………………………2 |  | **If No, skip to 40** |
| **38.** | Which Antibiotics were given? | IV ceftriaxone……………….…….1  IV Metronidazole………………….2  Oral Erythromycin…………………3  Others (Specify)…………………...4 |  |  |
| **39.** | What was the indication for the Antibiotics? | Prophylaxis for PROM……….…………………….1  Treatment for bacterial infections ……………………………………..2  Specify the infection……………………………. |  |  |
| **40.** | Were you given drugs to delay the labour? | No………………………………1  Yes………………………………2 |  | **If No, skip to 43.** |
| **41.** | Which of the following was given? | Magnesium sulphate…..…………..1  Nifedipine………………...………..2  Salbutamol…………………………3  Others (specify)……………………4 |  |  |
| **42.** | For how long did you receive the tocolytics? | <1 day……………………………..1  1-<3 days………………….………2  >3 days……………………………3 |  |  |

**PART B: MATERNAL AND FETAL OUTCOMES OF PRETERM LABOUR**

| **Question**  **Number** | **Questions** | **Response** |  | **Skip to** |
| --- | --- | --- | --- | --- |
| **43.** | **Maternal Outcome on discharge**:  Alive | No….................................................1  Yes… ...............................................2 |  |  |
| **44.** | Days spent in hospital. | < 24 hours………..………………...1  1<2 days……………………………2  2<3 days……….…………………...3  3< 7 days………………...…………4  >7 days……………...………..…….5 |  |  |
| **45.** | Did mother get any complications while in hospital? | No………………………………….1  Yes…..…………………………….2 |  | **If No. skip to 48.** |
| **46.** | Which complications? | Chorioamnionitis……………………1  Local wound infection……….……..2  Burst Abdomen…………………..…3  Pelvic Abscess…………………...…4  Puerperal Sepsis…………………….5  Postpartum hemorrhage…..………...6 |  |  |
| **47.** | Cause of death. | Sepsis……………………..……..….1  Hemorrhage..………………...….…..2  Anesthetic complications……..….....3  Others…………………………..…...4 |  |  |
|  | **Fetal Outcome** |  |  |  |
| **48.** | Was the baby admitted in the SCU post-delivery? | No…………………………………...1  Yes….………………………………2 |  | **If No, skip to 51.** |
| **49.** | How long was the baby admitted in the SCU? | <24 hours……...……………………1  1-<2 days……………………………2  2<3 days………………….…………3  3-7 days……………….……..……...4  >7 days…………………...…………5 |  |  |
| **50.** | What was the indication for admission in SCU? | Prematurity…………………...……..1  Neonatal Sepsis………………..……2  Seizure disorders…………………....3  Asphyxia (HIE)……………………..4  Congenital Anomalies……………....5  RDS………………………………...6  Trauma related…………………..….7 |  |  |
|  | **Outcome at Discharge** |  |  |  |
| **51.** | Alive | No…………………………………...1  Yes….………………………………2 |  |  |
| **52.** | If No, Was it any of the following? | FSB………………...……………...…..1  MSB…...………………..…………….2  ENND…………………………………3 |  |  |

**RESULTS:**

**Descriptive statistics**

The participant age was normally distributed, mean 26.0 SD±5.8, with the youngest being 14 years and the oldest 42 years. Median gravidity was 2.0, with interquartile range of 3.2. Most participants (64.0%) were either unemployed or housewives. HIV prevalence was 6.4%.

14% of the mothers had at least one previous scar. 2.3% of the mothers had previous operations on the cervix; all of them due ensure retention of the pregnancy to term. 85.5% of the mothers had live babies as the outcome of the previous pregnancy. However, 37% of the live babies were premature babies.

258/359 (71.8%) of the normal deliveries had preterm labor.

Caesarean section rates were five times higher in the group that had no preterm labor compared to mothers who had pre-term labor (3.7% vs. 15.6%).

50% of the mothers had history or drainage of liquor before onset of labor (PROM) and all of them had preterm birth.

Incidence of perinatal mortality is 77.8% 95% CI 73.4 – 81.7)


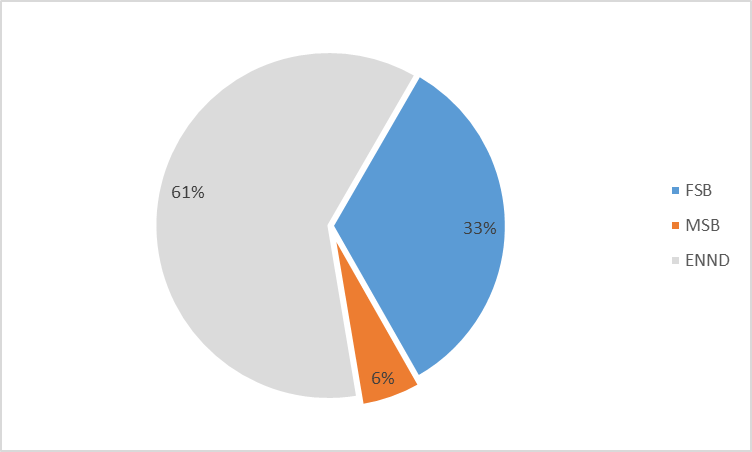


*Pie chart showing perinatal mortality by type*

**Table 1a: Baseline characteristics of 392 mothers at Kawempe National Referral Hospital, Kampala**

| Variable | Baby Alive (n=87)  n(%) | Baby Dead  (n=305)  n(%) |  | p-value |
| --- | --- | --- | --- | --- |
| **Age**  < 18 years  18 – 24 years  25 – 35 years  > 35 years | 5 (5.7)  39 (44.8)  31 (35.6)  12 (13.8) | 12 (3.9)  125 (41.0)  132 (43.3)  36 (11.8) |  | 0.589 |
| **Occupation**  Employed  Unemployed/housewife | 35 (40.2)  52 (59.8) | 106 (34.7)  199 (65.3) |  | 0.348 |
| **Does the participant smoke?**  Yes  No | 9 (10.3)  78 (89.7) | 43 (14.1)  262 (85.9) |  | 0.363 |
| **Monthly Income**  **<**50,000 UGX  50,000 - <500,000/=  500,000 - <1,000,000  >1,000,000 | 4  24  6  1 | 29  70  7  0 |  | 0.415 |
| **Gravidity**  Prime gravida  Gravida 2 – 3  Gravida ≥ 4 | 26 (29.9)  37 (42.5)  24 (27.6) | 93 (30.5)  113 (37.0)  99 (32.5) |  | 0.591 |
| **HIV Status**  Positive  Negative | 3 (3.4)  84 (96.6) | 22 (7.2)  283 (92.8) |  | 0.203 |
| **Labor pains before admission**  No  Yes | 29 (33.3)  58 (66.7) | 93 (30.5)  212 (69.5) |  | 0.614 |
| **Number of previous scars**  None  One  Two or more | 74 (85.1)  9 (10.3)  4 (4.6) | 263 (86.2)  32 (10.5)  10 (3.3) |  | 0.843 |
| **History of drainage of liquor**  Yes  No | 19 (21.8)  68 (78.2) | 85 (27.9)  220 (72.1) | 1.0  0.81 (0.50 – 1.31) | 0.261 |
| **Duration of drainage**  <24 hours  24<48hours  2-7 days  >7days | 19 (40.3)  18 (38.3)  5 (10.6)  5 (10.6) | 79 (53.0)  57 (38.3)  9 (6.0)  4 (2.7) | 1.0  0.76 (0.37 – 1.58)  0.43 (0.13 – 1.44)  0.92 (0.05 – 0.78) | -  0.464  0.172  **0.022** |
| **Color of liquor**  Clear  Light meconium  Thick meconium | 40 (85.1)  7 (14.9)  0 (0.0) | 129 (86.6)  18 (12.1)  2 (1.3) | 1.0  0.78 (0.31 – 2.04)  1.0 | -  0.638  - |
| **Smell of liquor**  Offensive  Not offensive | 6 (12.8)  41 (87.2) | 16 (10.3)  133 (89.7) | 1.0  1.22 (0.45 – 3.31) | -  0.701 |
| **Outcome of previous pregnancy**  Live Premature  Live full term  FSB  MSB  ENND  Congenital anomalies | 30  29  3  1  0  0 | 57  115  24  3  8  1 |  | **0.036** |

**Table 1b: Baseline characteristics of 392 mothers at Kawempe National Referral Hospital, Kampala**

| Variable | Baby Alive (n=87)  n(%) | Baby Dead  (n=305)  n(%) |  | p-value |
| --- | --- | --- | --- | --- |
| **Fever prior to admission**  No  Yes | 69 (79.3)  18 (20.7) | 257 (84.3)  48 (15.7) | 1.0  0.71 (0.39 – 1.31) | 0.276 |
| **Comorbidity at admission**  Malaria  UTI  Others | 7 (38.9)  8 (44.4)  3 (16.7) | 16 (33.3)  22 (45.8)  10 (20.8) | 1.0  1.20 (0.36 – 4.00)  1.45 (0.30 – 6.98) | -  0.763  0.647 |
| **History of vaginal bleeding**  No  Yes | 62 (71.2)  25 (28.8) | 204 (66.9)  101 (33.1) | 1.0  1.23 (0.73 – 2.07) | -  0.440 |
| **Labor medically induced**  **No**  **Yes** | 77 (88.5)  10 (11.5) | 250 (82.0)  55 (18.0) | 1.0  1.69 (0.82 – 3.48) | 0.148 |
| **Speculum exam done**  Yes  No | 16 (18.4)  71 (81.6) | 61 (20.0)  244 (80.0) | 1.0  0.90 (0.49 – 1.66) | 0.739 |
| **Digital exams per day**  None  1 – 2  3 – 4  ≥ 5 | 35 (40.3)  38 (43.7)  13 (14.9)  1 (1.1) | 133 (43.6)  148 (48.5)  23 (7.5)  1 (0.4) | 1.0  1.02 (0.61 – 1.71)  0.46 (0.21 – 1.01)  0.26 (0.02 – 4.31) | 0.925  0.053  0.349 |
| **Number of babies**  Singleton (one)  More than one baby  Not sure | 69 (79.3)  14 (16.1)  4 (4.6) | 249 (81.6)  41 (13.4)  15 (4.9) |  | 0.819 |
| **Drugs given to delay labor?**  No  Yes | 62 (71.3)  25 (28.7) | 214 (70.2)  91 (29.8) |  | 0.843 |
| **Mode of delivery**  SVD  Assisted VD  Emergency caesarian | 74 (85.1)  1 (1.1)  12 (13.8) | 285 (93.4)  3 (1.0)  17 (5.6) |  | **0.035** |
| **From decision to delivery**  < 24 hours  1<2 days  2<3 days  3< 7 days  >7 days | 56 (64.4)  22 (25.3)  6 (6.9)  1 (1.1)  2 (2.3) | 202 (66.2)  73 (23.9)  11 (3.6)  15 (4.9)  4 (1.3) |  | 0.335 |
| **Were corticosteroids given**  No  Yes | 35 (40.2)  54 (59.8) | 154 (50.5)  151 (49.5) | 1.0  0.60 (0.37 – 0.97) | **0.039** |
| **Were antibiotics given**  No  Yes | 37 (42.5)  50 (57.5) | 154 (50.5)  151 (49.5) | 1.0  0.72 (0.45 – 1.17) | 0.190 |
| **Duration of hospital stay**  < 24 hours  1<2 days  2<3 days  3< 7 days  >7 days | 14 (16.1)  21 (24.1)  11 (12.6)  26 (29.9)  15 (17.2) | 75 (24.6)  119 (39.0)  33 (10.8)  59 (19.3)  19 (6.2) |  | **0.001** |

*
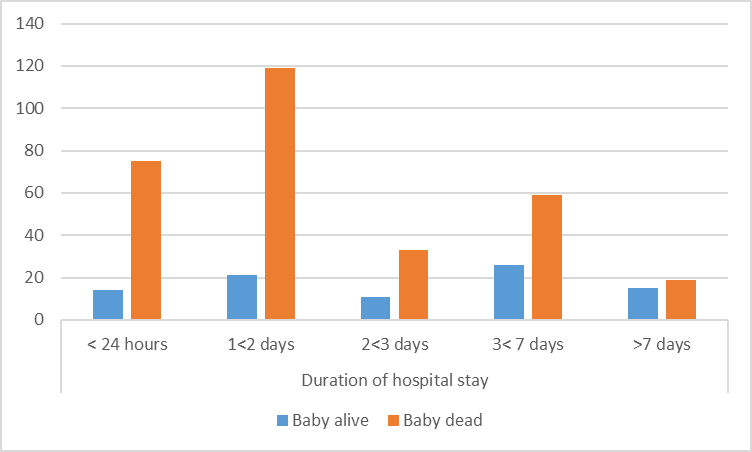
*

**Table 2: Comparison of Obstetric characteristics and Perinatal Mortality among 392 women who had Preterm delivery between 24 to 28 weeks of Gestation at Kawempe National Referral Hospital, Kampala, Uganda**

| **Variable** | Baby Alive (n=87)  n(%) | Baby Dead  (n=305)  n(%) | OR (95% CI) | p-value |
| --- | --- | --- | --- | --- |
| **Outcome of previous pregnancy**  Live Premature  Live full term  FSB  MSB  ENND  Congenital anomalies | 30  29  3  1  0  0 | 57  115  24  3  8  1 | 1.0  **2.09 (1.14 – 3.80)**  **4.21 (1.17 – 15.12)**  1.58 (0.16 – 15.84)  1.0 (-)  1.0 (-) | **0.016**  **0.028**  0.698  -  - |
| **Mode of delivery**  SVD  Assisted VD  Emergency C/S | 74 (85.1)  1 (1.1)  12 (13.8) | 285 (93.4)  3 (1.0)  17 (5.6) | 1.0  0.78 (0.08 – 7.60)  **0.38 (0.17 – 0.80)** | -  0.830  **0.012** |
| **Corticosteroids given**  No  Yes | 35 (40.2)  54 (59.8) | 154 (50.5)  151 (49.5) | 1.0  **0.60 (0.37 – 0.97)** | -  **0.040** |
| **Duration of hospital stay**  < 24 hours  1<2 days  2<3 days  3< 7 days  >7 days | 14 (16.1)  21 (24.1)  11 (12.6)  26 (29.9)  15 (17.2) | 75 (24.6)  119 (39.0)  33 (10.8)  59 (19.3)  19 (6.2) | 1.0  1.06 (0.51 – 2.21)  0.56 (0.23 – 1.56)  **0.42 (0.20 – 0.88)**  **0.24 (0.10 – 0.57)** | -  0.881  0.201  **0.022**  **0.001** |
| **Tocolytics given**  No  Yes | 62 (71.3)  25 (28.7) | 214 (70.2)  91 (29.8) | 1.0  1.05 (0.62 – 1.78) | -  0.843 |
| **Mode of delivery**  SVD  Assisted VD  Emergency C/S | 74 (85.1)  1 (1.1)  12 (13.8) | 285 (93.4)  3 (1.0)  17 (5.6) | 1.0  0.78 (0.08 – 7.60)  0.36 (0.17 – 0.80) | -  0.830  **0.012** |
| **Number of babies**  Singleton (one)  >1 baby  Not sure | 69 (79.3)  14 (16.1)  4 (4.6) | 249 (81.6)  41 (13.4)  15 (4.9) | 1.0  0.81 (0.42 – 1.57)  1.04 (0.33 – 3.23) | -  0.537  0.947 |

**Other statistics:**

- 2.6% (n=10) of the mothers developed complications.
- Most of the babies (n=231, representing 58.9%) required SCU admission, with 31% of them spending more than 7 days.
- Delivery by Caesarean Section was protective from occurrence of perinatal mortality OR=0.37 (95% CI 0.17 – 0.80, p-value=0.012) in comparison with the group that had normal vaginal delivery.
- HIV infection increased the risk of perinatal mortality. However, this was not statistically significant; OR=2.18 (95% 0.63 – 7.48, p-value=0.213). Similarly, there was no association between maternal mortality and HIV status (p-value=0.379).
- For this population, the Infant Mortality Rate (Crude Death Rate) is 77,806 per 100,000. SVD group had CDR of 79,387 per 100,000 while C/Section group had CDR of 58,620 per 100,000. Overall maternal mortality rate was 2,806 per 100,000. In the SVD group, CDR was 2,785 per 100,000 compared to zero in the Caesarean Section group.
- Only two complications were reported: Local wound infection (40%) and postpartum haemorrhage (60%). All cases of PPH were reported in SVD group.
- 58.9% of the babies were admitted to SCU. SVD accounted to 89.1% of the admissions to SCU. 193/231 (93.1%) of the admissions to SCU were due to prematurity. The other indications were Asphyxia (6.1%) and Respiratory Distress Syndrome (0.9%). SVD accounted for most admissions to SCU (89.2%), followed by Caesarean Section (9.5%).

**Multivariate Analysis**

Caesarean Section (compared to spontaneous vaginal delivery) OR=0.34, 95% CI 0.14 – 0.82, p-value=0.017

Receiving steroids OR=0.57, 95% CI 0.33 – 0.98, p-value=0.040

Doing 3 – 4 digital exams per day (the reference group is one where no digital exam was done), OR=0.41, 95% 0.18 – 0.91, p-value=0.028 (Table 3).

**Table 3: Multivariate association between Preterm Labor and Independent Variables for mothers delivering at Kawempe National Referral Hospital, Kampala, Uganda**

| **Variable** |  | |
| --- | --- | --- |
|  | **OR (95% CI)** | **p-value** |
| More than one baby | 15.45 (2.00 – 119.53) | <0.001 |
| Presence of fever | 4.03 (95% CI .23 – 13.23) | 0.002 |
| Drainage of liquor | 0.16 (0.03 – 0.87) | 0.034 |


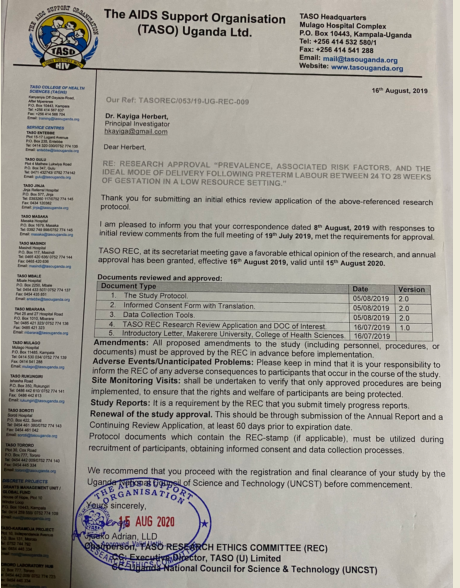

Supplement: S1 File — (DOCX) [file pone.0254801.s001.docx]
